# A Novel Heterometallic Ring {Cr_5_Ni_3_} and New {Cr_6_Co_2_} and {Cr_6_Zn_2_} Rings

**DOI:** 10.1002/chem.70991

**Published:** 2026-04-16

**Authors:** Abdulelah Alsuhaymi, Niklas Geue, Adam Brookfield, Grigore A. Timco, Selena J. Lockyer, Gareth D. Smith, George F. S. Whitehead, Perdita E. Barran, David Collison, Richard E. P. Winpenny

**Affiliations:** ^1^ Department of Chemistry The University of Manchester Manchester UK; ^2^ Department of Chemistry Faculty of Science Islamic University of Madinah Madinah Saudi Arabia; ^3^ Michael Barber Centre For Collaborative Mass Spectrometry Manchester Institute of Biotechnology Department of Chemistry The University of Manchester Manchester UK

## Abstract

A new heterometallic ring, [Me_4_N][(Me_4_N)_2_Cr_5_Ni_3_F_8_(BTFB)_16_] (BTFB = 3,5‐bis(trifluoromethyl)benzoate) is reported, templated about tetramethylammonium (Me_4_N)^+^. This ring features five Cr(III) centers and three Ni(II) centers at the vertices of an octagon. Each edge of the octagon is bridged by internal fluoride and two external BTFB ligands. In the crystal structure, two of the three ammonium cations are at the center of the octagonal ring, while the third cation is outside. X‐ray single crystal diffraction does not distinguish between five possible isomers, but a single isomer, with nickel sites at the 1, 3, and 6 positions of the octagon, can be deduced by a combination of collision‐induced dissociation mass spectrometry (CID‐MS), and EPR spectroscopy and magnetometry, which show a ground state spin of ^1^/_2_. The same reaction carried out with cobalt(II) or zinc(II) in place of nickel(II) leads to ordered [(Me_4_N)_2_Cr_6_M_2_F_8_(BTFB)_16_] (M = Co or Zn) in which the divalent metals are placed at the 1,5‐positions in the metal octagon.

## Graphical abstract

A novel heterometallic ring containing five Cr(III) and three Ni(II) centers is reported. It forms an octagon and has a single isomer, as revealed by a combination of magnetic and EPR studies and collision‐induced dissociation mass spectrometry.

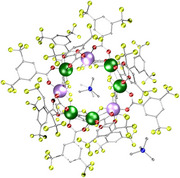

## 1 Introduction

The idea that a polymetallic structure can be directed through a templating ion or molecule is appealing since it opens the possibility of making multimetallic compounds using planned synthesis rather than relying on serendipity [1]. Saalfrank and Raymond both made important contributions to this idea. Saalfrank demonstrated that the ionic radius of the alkali metal ion template can be used to select hexa‐ or octa‐nuclear rings [2], and Raymond demonstrated that different templates can direct the nuclearity and shape of gallium clusters [3].

Several groups have made cyclic heterometallic rings in recent years, mostly featuring a mixture of 3d and 4f metals. For example, there are {Cr^III^_4_Dy^III^_4_} [4] and related {Fe^III^_4_Ln^III^_4_} (Ln^III^ = Dy^III^, Gd^III^, Y^III^) [5] rings by the Powell and Konar groups, respectively; there are multiple rings featuring Fe^III^ and Yb^III^ [6]; a family of nine‐metal rings {Fe^III^_6_Ln^III^_3_} {where Ln^III^ = Gd^III^ to Lu^III^, Y^III^) [7]; {Mn^III^_8_Ln^III^_8_} [8] and {Cr^III^_8_Ln^III^_8_} rings [9]; large {Co^II^_16_Ln^III^_24_} [10] and {Co^II^_36_Ln^III^_24_} rings [11]. Mostly these have been studied for their magnetic properties, but there is a recent report of a {Cd^II^_6_Yb^III^_6_} ring which shows near‐IR luminescence [12]. The 3d‐4f rings have a large advantage in characterization, as X‐ray crystallography identifies the sites occupied by the different metals unambiguously.

Heterometallic rings with the formula {Cr^III^_x_M^II^_y_} have attracted interest for their physical and structural properties [13, 14]. These rings have potential uses in quantum information processing [15] and as resists for electron beam lithography [16]. The first family of heterometallic rings, {Cr^III^_7_M^II^} **1‐M**, was reported in 2003 [17], and has since been widely studied with variations in metals, templates, fluorides, and carboxylates [18, 19, 20]. Divalent metals are rarely localised in the crystal structure, although this can occur if the metal has a large ionic radius or prefers a specific coordination geometry [21, 22].

Increasing the number of divalent metals in the ring brings the possibility of linkage isomers [23], which will also affect the physical behaviour of the heterometallic compound. In 2021, Alotaibi et al. reported complexes that contain localised divalent metals in the ring [24], with the ring templated about a tetramethylammonium cation. The general formula of this family of octametallic rings is [Me_4_N]_2_[Cr^III^_6_M^II^_2_F_8_(O_2_C*^t^* Bu)_16_] **2‐M**, where M = Zn, Mn, Ni, or Co. All metal ions have an octahedral coordination geometry, and divalent metals are found in positions 1 and 5 of the ring [24]. More recently, using the carboxylate 3,5‐bis(trifluoromethyl)benzoate (BTFB) the complexes [Pr_2_NH_2_][(Pr_2_NH_2_)Cr_6_M_2_F_8_(BTFB)_16_] **3‐M** were made, where M = Ni(II), Zn(II) or Co(II) [25]. In **3‐M**, the divalent metals are disordered around the octagon, with the physical properties showing that at least two isomers are present.

These results with Me_4_N^+^ as the templating cation or BTFB^−^ as the carboxylate both give {Cr^III^_6_M^II^_2_} rings rather than {Cr^III^_7_M^II^} rings, which made us examine what would happen if both Me_4_N^+^ and BTFB^−^ were used. Here, we present the first example we have of a {Cr^III^_5_Ni^II^_3_} ring [Me_4_N][(Me_4_N)_2_Cr_5_Ni_3_F_8_(BTFB)_16_] **4**, and single‐isomer {Cr^III^_6_M^II^_2_} rings [(Me_4_N)_2_Cr^III^_6_M^II^_2_F_8_(BTFB)_16_] **5‐M**, where M = Zn, or Co. Characterization of **4** as a single isomer required an unusual combination of advanced mass spectrometry, magnetic and EPR studies.

## 2 Results

### 2.1 Synthesis

The reagents, tetramethylammonium hydroxide, hydrated chromium fluoride, the appropriate metal carbonate, and BTFBH were mixed in 1,2‐dichlorobenzene and heated to 140°C, and stirred for 50 h (experimental details in Supporting Information). The reaction was allowed to cool to room temperature, and MeCN was added to cause precipitation. The green product was collected by filtration and extracted with acetone. The solution was then mixed with toluene, evaporated to dryness under reduced pressure, and washed with toluene and hexane. Crystals of **4** or **5‐M** were obtained from toluene in moderate yield.

### 2.2 Mass Spectrometry

**FIGURE 1:**
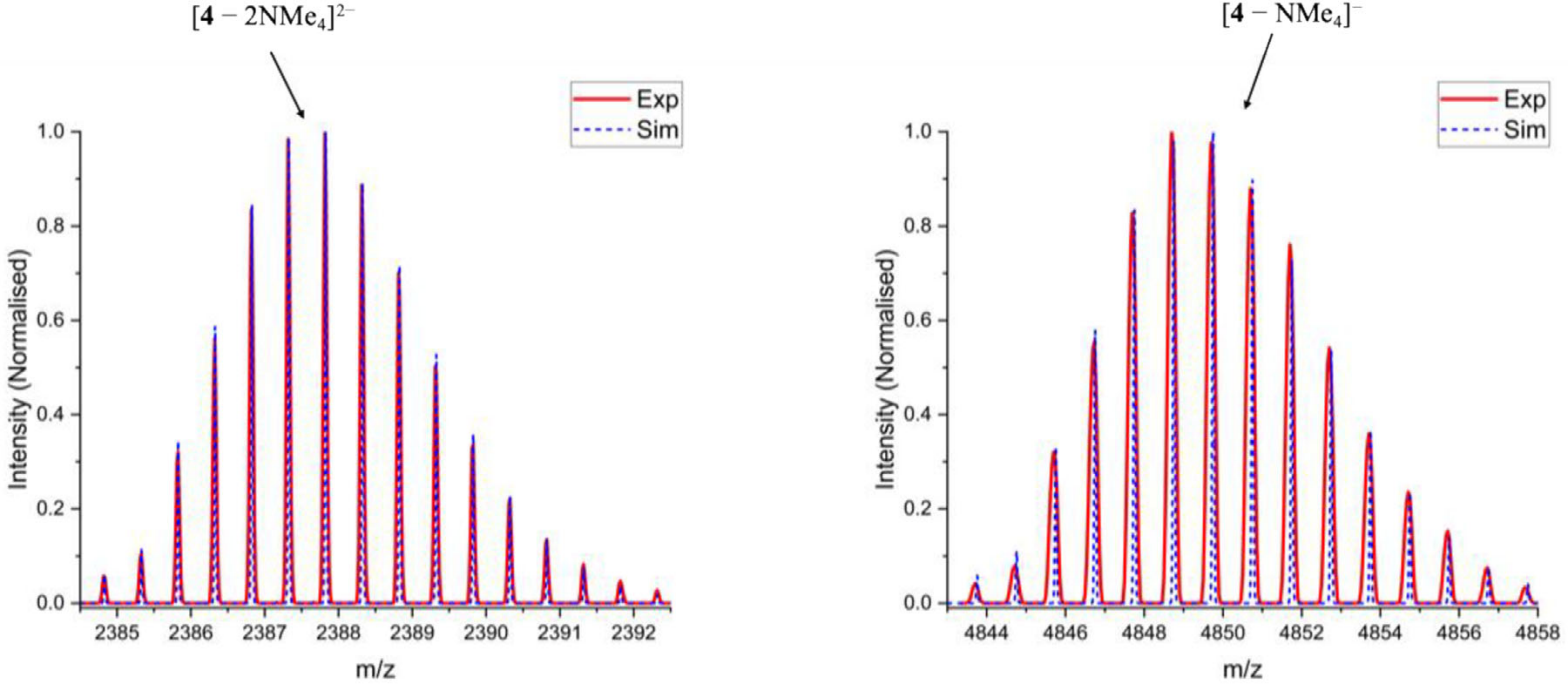
The isotopic distributions in the negative mode ESI‐MS spectrum of **4**, due to the loss of two cations (left) and one cation (right). Experimental data are shown in red, while the simulation is in blue.

Compound **4** was analyzed using ESI‐MS. The data revealed multiple unknown ions in the positive mode with low overall signal intensity (Figure S1). In the negative mode, two observable distributions were identified: one distribution is due to a dianion and showed the loss of two cations [**4**−2 Me_4_N]^2−^, and the second distribution is a monoanion and showed the loss of one cation [**4**−Me_4_N]^−^ (Table 1, Figure 1; full negative mode MS in Figure S2).

**TABLE 1:** The values of *m*/*z* in ESI‐MS for the observed and calculated peaks for **4** and **5‐M**.

Fragment	Observed peaks (calculated)/ amu		
	4	5‐Zn	5‐Co
Singly charged ions	4849.68 (4850.05)	4782.19 (4782.65)	4769.61 (4769.72)
Doubly charged ions	2387.82 (2387.95)	2354.06 (2354.26)	2347.78 (2347.79)

**FIGURE 2:**
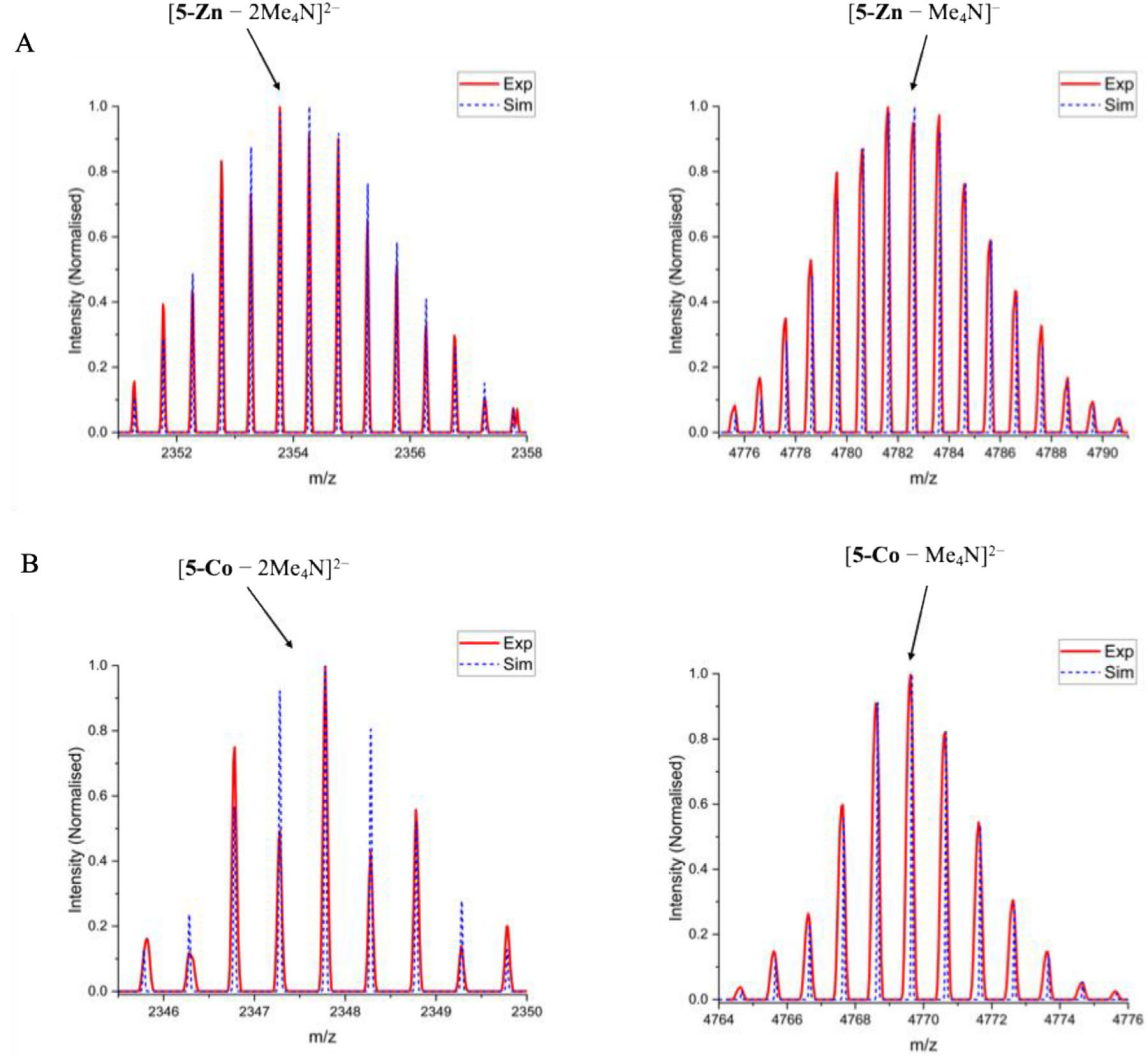
The negative mode ESI‐MS spectra of **5‐M**, (A) **5‐Zn** and (B) **5‐Co**. For both **5‐Zn** and **5‐Co**, the spectrum shows two peaks that are responsible for the loss of two cations (left) and one cation (right). Experimental data are shown in red, while the simulation is in blue.

**FIGURE 3:**
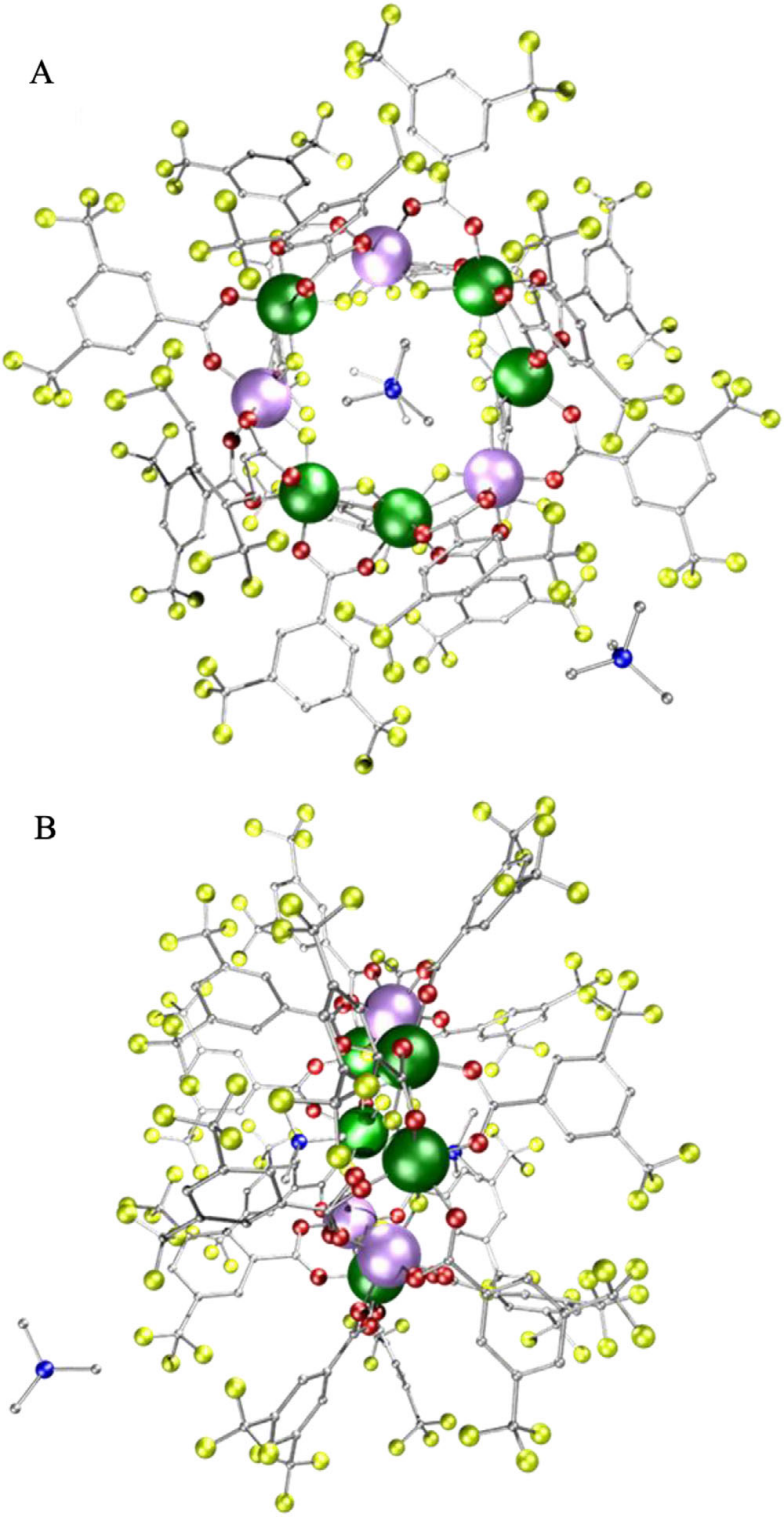
The crystal structure of **4** in the crystal, shown as the 1,3,6‐isomer, viewed: (A) perpendicular to the plane of the metals and (B) side view. Colors: Cr, green; Ni, purple; F, yellow; O, red; N, blue; C, grey. H‐atoms omitted for clarity. The external Me_4_N cation is shown.

In previous studies that gave **2‐M** and **3‐M**, we observed that cobalt, nickel, and zinc produced similar ESI‐MS spectra [24, 25]. However, here we see a difference between nickel, compound **4**, and zinc and cobalt in **5‐M**, which exhibit different ESI‐MS data than **4** (Table 1 and Figure 2). For all three divalent metals, we see distributions for mono‐ and di‐anions. However, for cobalt and zinc, the mass and isotopic distributions for the mono‐ and di‐anion are consistent with the octametallic rings [(Me_4_N)_2_Cr^III^_6_M^II^_2_F_8_(BTFB)_16_] **5‐M**, where M = Zn, or Co, whereas these data for **4** are consistent with [Me_4_N][(Me_4_N)_2_Cr_5_Ni_3_F_8_(BTFB)_16_]. The ESI‐MS data for both **5‐M** compounds show multiple unknown ions in positive mode, accompanied by low overall signal intensity (Figures S3 and S4). In the negative mode for **5‐M**, there were two observable distributions: the first due to the loss of two cations [**5‐M**−2 Me_4_N]^2−^, and the second due to the loss of one cation [**5‐M**−Me_4_N]^−^ (Figure 3; full negative mode data in Figures S5 and S6). These findings for the **5‐M** are similar to those observed for the **3‐M** [25].

### 2.3 Crystallography

Single crystals of **4** were grown through the slow evaporation of a mixed solvent solution containing either acetone/toluene or THF/toluene. The compound crystallizes in the tetragonal space group P4 with half of the metal octagon in the asymmetric unit (Table S1). The refined structure reveals the formation of a single octametallic ring, with two tetramethylammonium cations positioned above and below the mean plane of the ring within the ring cavity (Figure 3a). The bulky fluorinated ligands result in inefficient crystal packing, making crystallography challenging. The third cation needed for charge balance is disordered and found within the large lattice voids created by the arrangement of the rings.

Each M…M edge of **4** is internally connected by one fluoride and externally linked by two carboxylates, demonstrating a clear relationship to previous octametallic rings. One carboxylate group on each edge lies in the plane of the metal octagon, occupying an equatorial position, while the other carboxylate group is oriented perpendicular to the metal plane, taking on an axial position. The axial carboxylates alternate positions above and below the plane as one moves around the metal octagon (Figure 3b). The two NMe_4_^+^ cations close to the ring lie in pockets between these axial carboxylate groups (Figure 3b).

**FIGURE 4:**
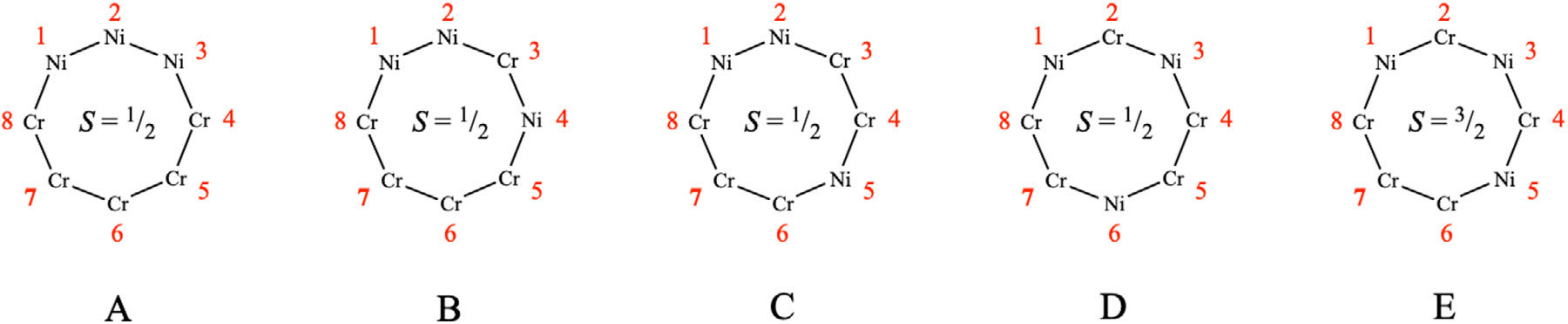
Different isomers of **4** might exist regarding the three Ni(II) metal positions, and assuming antiferromagnetic coupling, the ground state of each isomer is given.

The presence of three nickel(II) metals in an octagon may result in isomerization, leading to the possibility of five distinct isomers: 1,2,3‐, 1,2,4‐, 1,2,5‐, 1,3,6‐, or 1,3,5‐ (Figure 4). We have previously seen multiple isomers in related chemistry [17]. The high symmetry of the structure means that each metal site in the structure is mixed between Cr^III^ and Ni^II^, and it is not possible to localize the divalent sites. The metric parameters (Table 2) show metal‐oxide and metal‐fluoride bond lengths that span the range of both metals. The M….M….M angles at the corners of the octagon range from 130.81(2) to 138.67(2)*°*.

**TABLE 2:** Selected bond lengths(Å) and M…M…M angles(°) in **4**, **5‐Co** and **5‐Zn**.

	4	5‐Co	5‐Zn
Bond lengths/Å			
M^II^–O	1.901(14)–2.101(13)	2.012(5)–2.082(16)	2.00(2)–2.09(2)
Cr^III^–O		1.925(4)–2.017(18)	1.929(6)–2.005(6)
M^II^–F	1.850(18)–1.987(19)	1.984(15)–2.049(15)	2.02(6)–2.09 (5)
Cr^III^–F		1.867(13)–1.954(11)	1.87(4)–1.930(5)

M…M…M angle/°			
At M^II^	130.81–138.67	129.01 and 129.24	126.0 and 126.2
At Cr^III^		133.65–138.08	134.1–138.3

Therefore, the crystallography does not distinguish between the five possible isomers. The magnetic and spectroscopic properties of four of the five isomers should also be similar, with *S* = 1/2 ground states. Isomer E would have an *S* = 3/2 ground state, and therefore, we should be able to deduce whether it is present from a magnetic study.

**FIGURE 5:**
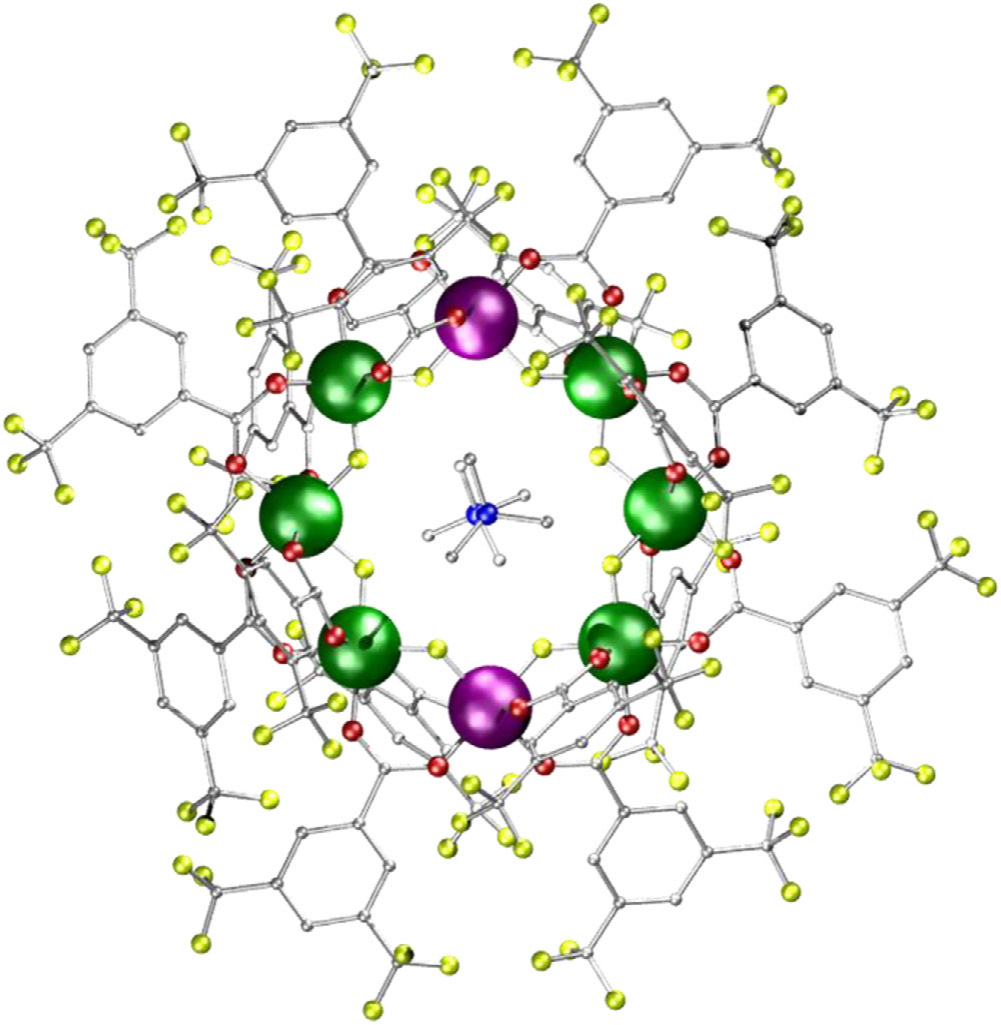
The crystal structure of **5‐Co** in the crystal viewed perpendicular to the plane of the metals. Colors as Figure 3, plus Co, purple.

The crystal structures of **5‐Co** and **5‐Zn** are disordered, severely so in the case of **5‐Zn**. In both cases, a metal octagon is found, with each edge bridged by a fluoride and two BTFB ligands (Figure 5). The divalent metal sites are at the 1,5‐position of the metal octagon based on metric parameters: the M^II^‐O and M^II^‐F bond lengths are longer than the Cr^III^‐O and Cr^III^‐F bond lengths (Table 2); the disorder in **5‐Zn** leads to overlap in the ranges for the metal‐oxide bonds, but the trend is clear, and supported by spectroscopy (see below). Secondly, the M…M…M angle is more acute at the divalent sites. The NMe_4_^+^ cations sit above and below the plane of the metal octagon. The structure of **5‐Zn** is shown in Figure S7. A noticeable feature of these structures is the presence of toluene solvate molecules near the exterior of the heterometallic rings. It appears there is a favorable interaction between the fluorinated carboxylates and the solvate. This was observed in other studies using this carboxylate [25, 26, 27, 28, 29, 30].

### 2.4 Ion Mobility Mass Spectrometry and Collision‐Induced Dissociation Studies of 4

As **4** may contain a mixture of five isomers of the {Cr_5_Ni_3_} ring (Figure 4), we used advanced mass spectrometry techniques to identify the number and type of linkage isomers, focusing on the negative ion mode based on the ESI‐MS results above.

The dianion [**4**–2 NMe_4_]^2−^ was *m/z*‐selected in a quadrupole mass filter and subjected to cyclic ion mobility spectrometry (cIMS) [31]. In cIMS, ions are separated according to the time they require to traverse a nitrogen filled cyclic drift cell (“arrival time”), when guided by an electric field of travelling waves [32]. Separation is achieved through differences in the interactions with the mobility gas. Larger and more extended ions exhibit more collisions and take more time to travel through the cIMS cell, whereas smaller, compact structures are faster as they experience less collisions. We have previously used cIMS to assess the topology of polymetallic complexes from the same family as **4** based on their arrival times and packing densities [32, 33, 34, 35, 36, 37], as well as to resolve minor differences induced by adduct ions (H^+^, Na^+^, K^+^, Cs^+^) in such metallosupramolecules [38].

**FIGURE 6:**
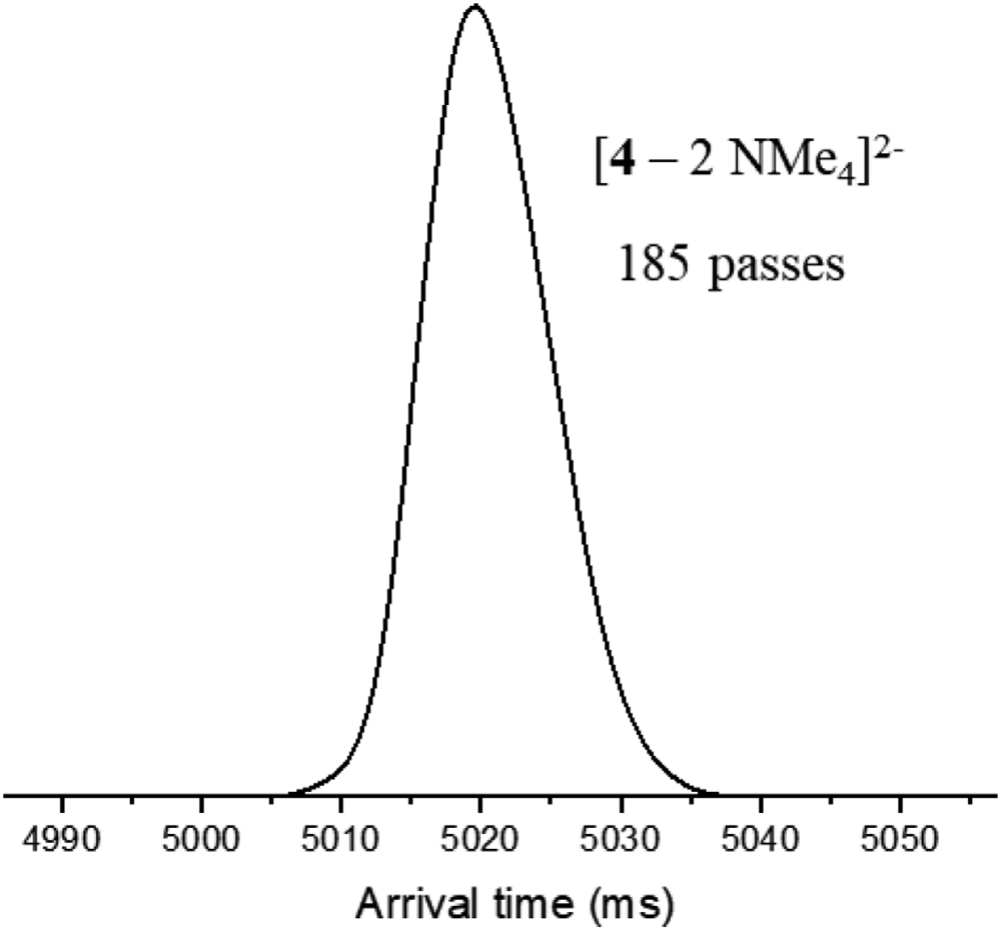
Separation of [**4**–2 NMe_4_]^2−^ over 185 passes in a cyclic ion mobility device [1]. Data were smoothed and presented as a unimodal distribution, suggesting the presence of a single linkage isomer.

The [**4**–2 NMe_4_]^2−^ was subjected to 185 passes in the cIMS cell (Figure 6). The resulting arrival time distribution is unimodal, suggesting the presence of a single isomer. It can be argued that the differences in Cr^III^ and Ni^II^ distribution within **4** might not have a detectable impact on the ion mobility; however, cIMS has been shown in the past to resolve minor structural differences. For example, anomers and open‐ring forms of pentasaccharides were resolved after 18 passes [39], and even diastereomers of M_4_L_6_ cages could be completely distinguished after 150 passes [40]. The absence of any separation for **4** after 185 passes, which is the software limit of the instrument, strongly suggests the presence of a single linkage isomer. There is a small asymmetry to this peak, and we cannot completely exclude this being due to a small amount of a second isomer.

**FIGURE 7:**
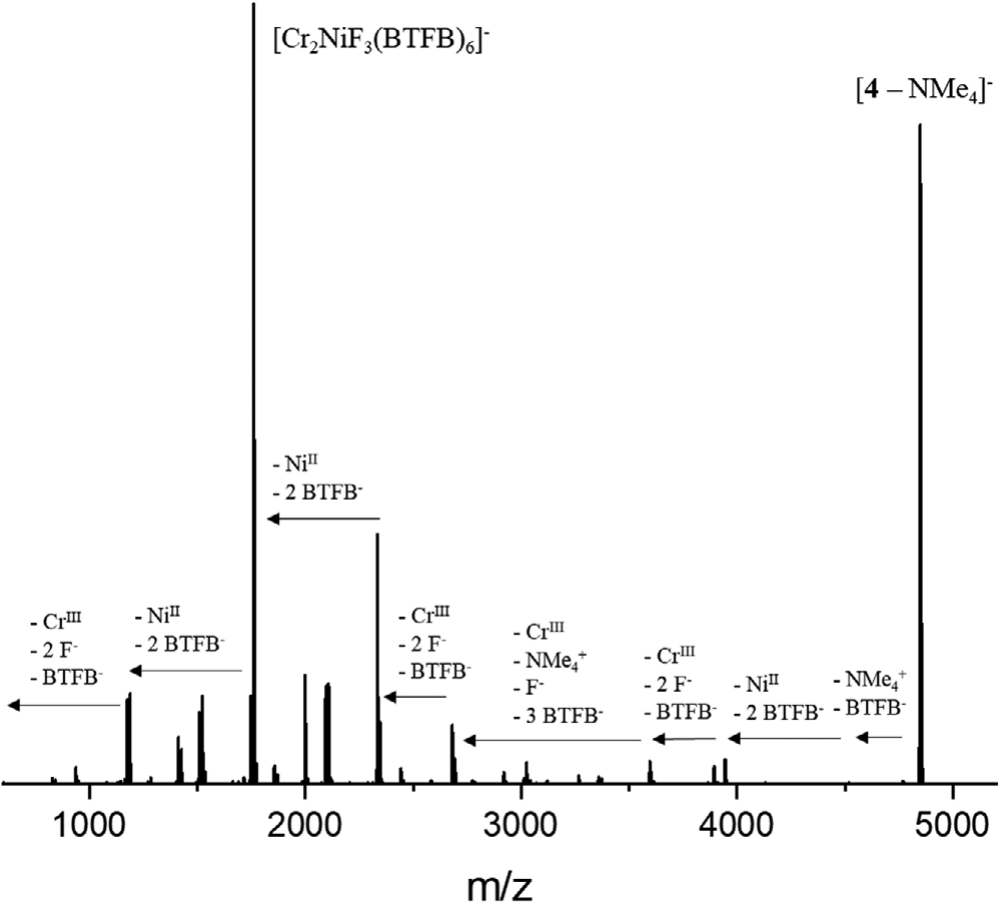
CID‐MS spectrum of [**4**–NMe_4_]^−^ (*m/z* = 4850) at collision energy in the laboratory frame *E*_lab_ = 200 eV. Fragmentation channels are diverse, and the most dominant one for each step is labelled.

We aimed to analyze the fragmentation pathways and stabilities of the ions [**4**–NMe_4_]^−^ and [**4**–2 NMe_4_]^2−^ using collision‐induced dissociation mass spectrometry (CID‐MS). This technique is uniquely qualified to investigate disassembly reactions and stabilities in the gas‐phase, where solvent molecules and counter ions are absent [35, 41, 42]. In CID‐MS, ions are subjected to collisions with an inert gas (here: nitrogen) at user defined‐energies, which usually leads to fragmentation. The dissociation of both ions proceeds *via* highly similar pathways, and we will focus on the disassembly of [**4**–NMe_4_]^−^. The fragmentation channels are complex, and we labelled the most dominant one for each step in Figure 7. In the first step, one NMe_4_^+^ and one BTFB^−^ ligand dissociate as the main fragmentation channel. This agrees well with our previous publications, where the first leaving group is always the loss of the noncovalently bound ammonium cation and a carboxylate ligand [33, 34, 43]. This is followed by a range of fragmentation channels, in which the most dominant exhibit the order of metal center loss as follows: Ni^II^, Cr^III^, Cr^III^, Cr^III^, Ni^II^, Ni^II^, Cr^III^. This order of metal losses is also found for the ion [**4**–2 NMe_4_]^2−^ (Figure S8).

As we have previously shown and discussed [44], the binding of Ni^II^ to the carboxylate and fluoride ligands is weaker than that of Cr^III^ due to Coulombic reasons, and hence the three Ni^II^ centers are the weakest points in the {Cr_5_Ni_3_} ring. This explains that the first observed metal loss corresponds to Ni^II^. After one metal center is lost, the ring opens, becoming a seven‐metal “horseshoe”, and the two adjacent metal centers are expected to be least strongly bound and hence to dissociate next. The three following dominant metal losses are then Cr^III^, which suggests that removal of a single Ni^II^ produces an open chain terminated at both ends by Cr^III^. In turn, this discounts any of the isomers where there are adjacent nickel sites, i.e., isomers **A**, **B**, and **C** (Figure 4). Magnetic and EPR studies show an *S *= 1/2 ground state (see below), which excludes isomer **E**, and therefore the CID‐MS suggests isomer **D**, i.e., the isomer with Ni^II^ in positions 1, 3, and 6 of the octagon. The most abundant fragment we see (Figure 7) is [Cr^III^_2_NiF_3_(BTFB)_6_]^−^ ion, which may be a metal triangle.

**FIGURE 8:**
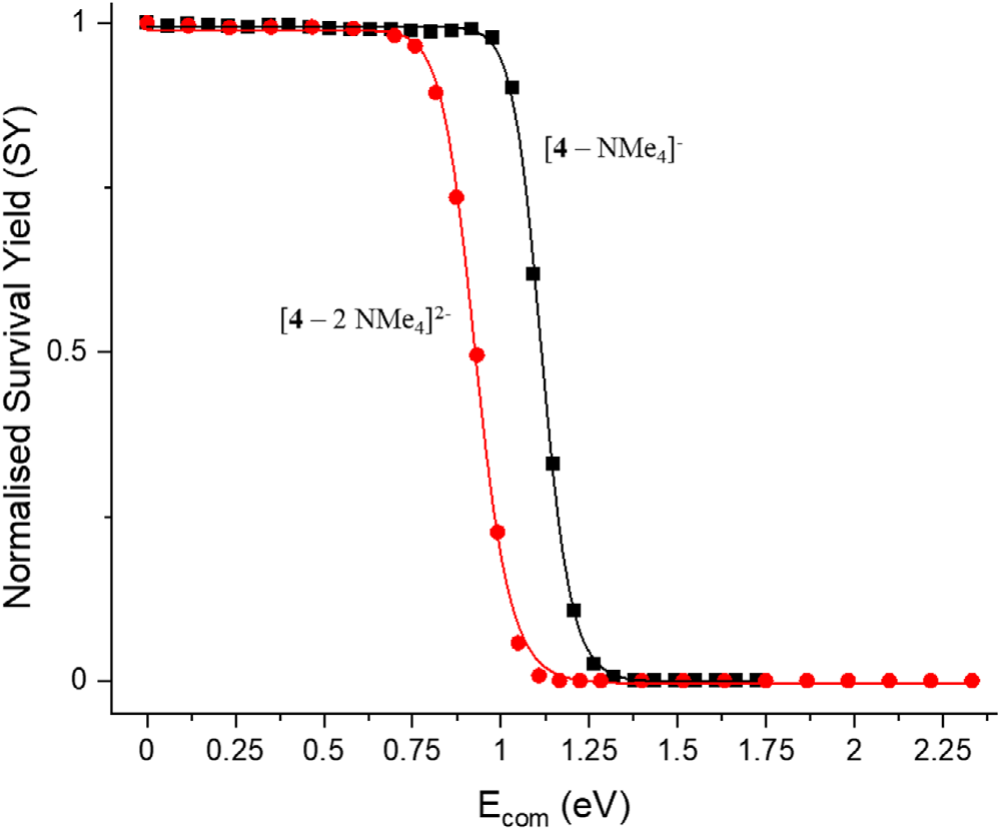
Survival yield versus collision energy in the center‐of‐mass frame (*E*_com_) of [**4**–NMe_4_]^−^ and [**4**–2 NMe_4_]^2−^, showing a higher stability of [**4**–NMe_4_]^−^ compared to [**4**–2 NMe_4_]^2−^.

We quantified the relative stabilities of the ions [**4**–NMe_4_]^−^ and [**4**–2 NMe_4_]^2−^, which both dissociate through similar fragmentation pathways as discussed above. By ramping the collision energy, we can measure the share of parent ion that survives the collision cell (“survival yield”, *SY*) at various energies (Figure 8). The energy (in center‐of‐mass frame, *E*_com_) at which 50% of the parent ion fragments (*SY* = 0.5) can be quantified through sigmoidal fitting curves, and this *E*_50_ value can be regarded as a relative measure of ion stability [45, 46, 47].

The resulting *E*_50_ values are (1.1155 ± 0.0011) eV for [**4**–NMe_4_]^−^ ion, and (0.9285 ± 0.0017) eV for [**4**–2 NMe_4_]^2−^. We previously measured the *E*_50_ value of the isostructural ion [Cr_6_Ni_2_F_8_(BTFB)_16_ + NH_2_^n^Pr_2_]^−^, where we found a 6% higher *E*_50_ value than for [**4**–NMe_4_]^−^ (same charge) and 27% higher than for [**4**–2 NMe_4_]^2−^ (same number of small ammonium cations) [25]. This suggests that {Cr_5_Ni_3_} is less stable than {Cr_6_Ni_2_}, in accordance with our previous results that indicate a destabilizing effect of divalent metals in polymetallic chromium rings. However, the size of the ammonium cation is different, which has previously been found to have an impact on the *E*_50_ value [32, 42]. In the absence of any ammonium cation, an *E*_50_ value of (1.100 ± 0.017) eV was found for [Cr_7_NiF_8_(BTFB)_16_]^−^, similar to that of [**4**–NMe_4_]^−^. This suggests that the *E*_50_ values are determined by a complex interplay of metal composition, ammonium ligands, and, as previously found for BTFB, possible inter‐ligand interactions [25, 44]. Overall, these preliminary studies support our previous proposition that CID‐MS is a valuable tool in understanding the bonding in polymetallic complexes.

### 2.5 Magnetic Studies

**FIGURE 9:**
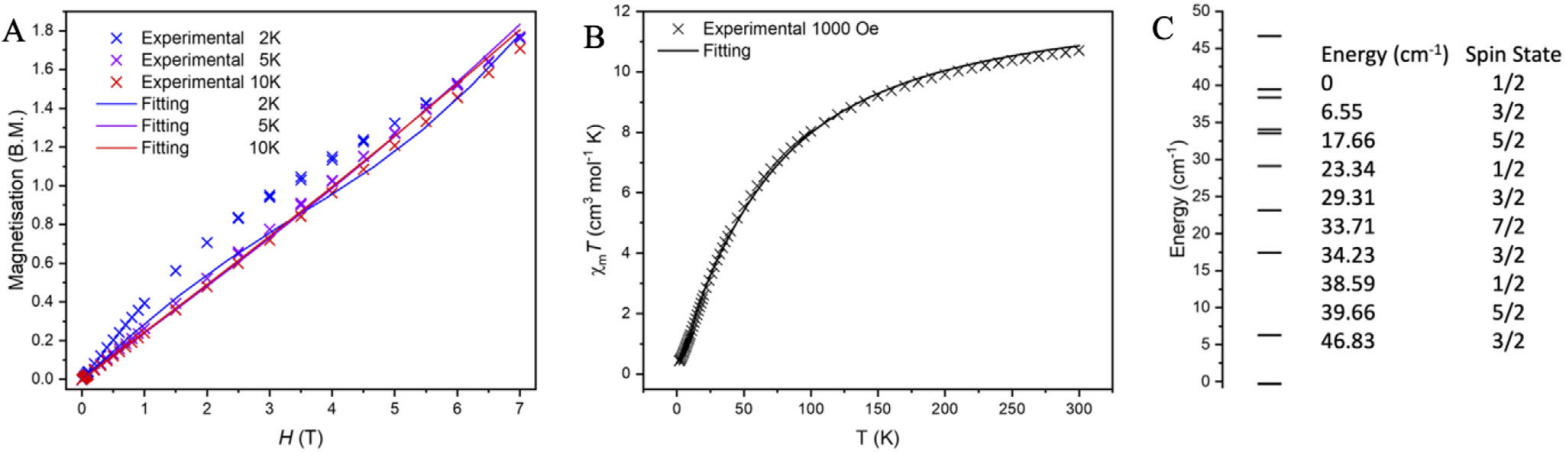
Magnetic studies of **4** fitted as isomer **D** (A) *M*(*H*) measured at 2, 5 and 10 K. (B) χ_*M*_ *T*(*T*) measured at 1000 Oe. In (A) and (B), experimental data are shown as crosses, simulation using the parameters in Table 3 is shown as a line. (C) The lowest energy states resulting from the simulation shown in (A) and (B).

The magnetic susceptibility (*χ*_M_) of compound **4** was measured in the temperature range of 2 to 300 K in a field of 1000 Oe. The value of *χ*_M_ *T* decreases as the temperature falls (Figure 9a), which is consistent with antiferromagnetic interactions between the metal centers, as expected compared with the first heterometallic wheel **1** [17], the single‐isomer rings **2‐M** [24], and the compounds with disordered metal positions in **3‐M** [25]. **4** comprises three Ni(II) and five Cr(III) ions. The presence of three Ni(II) centers in **4** suggests that there may be multiple isomers (Figure 4), with an *S* = 1/2 ground state for four of these isomers. While the X‐ray crystallography does not identify a single isomer, the CID‐MS narrows the alternatives down to the two isomers where there are no nickel sites adjacent to each other, i.e., isomers **D** and **E**. Isomer **E** has all the Ni^II^ sites in the same sub‐lattice, which would give a spin ground state *S *= 3/2. The magnetic studies and EPR spectroscopy confirm **E** is not seen.

The magnetization (*M*) for **4** was measured as a function of field at 2 K, 5 K, and 10 K (Figure 9b). Saturation of magnetization is not achieved at any temperature, suggesting the presence of low‐lying excited states. Given the results of the CID‐MS studies, the *χ*_M_ *T*(*T*) and *M*(*H*) data were fit simultaneously using the PHI software [48] for isomer **D**, giving the parameters summarized in Table 3. The result of this simulation is an *S* = ½ ground state with an *S* = ^3^/_2_ first excited state at 6.55 cm^−1^ (Figure 9c). The *J*_CrNi_ is found to be −10.73 cm^−1^, similar to that found for **3‐Ni**. *J*_CrCr_ is smaller than we usually find. The exchange values found are reasonable, but the data are not sufficient to determine them unambiguously, particularly as we have not included any zero‐field splitting parameters for the three Ni(II) sites, and these could be substantial. As other isomers are possible from the X‐ray and magnetic studies, we have also fitted the data to isomer **A**, which is the 1,2,3‐isomer (Figure S8).

**TABLE 3:** Spin Hamiltonian parameters (*J* in cm^−1^) used to simulate magnetic data measured for **4** and **5‐Zn**, with parameters for **3‐Ni** and **3‐Zn** included for comparison. A –2*J* coupling Hamiltonian is used in PHI [48], so negative *J* denotes antiferromagnetic coupling.

Compound	*J*_CrCr_	*J*_CrM_	*J*_CrMCr_	*g*
**4**	−3.30	−10.73	N/A	1.97^Cr^, 2.20^Ni^
**5‐Zn**	−4.48	N/A	−0.027	1.97^Cr^
**3‐Ni**	−4.60	−10.40	N/A	1.97^Cr^, 2.20^Ni^
**3‐Zn**	−4.60	N/A	−0.15	1.96^Cr^

**FIGURE 10:**
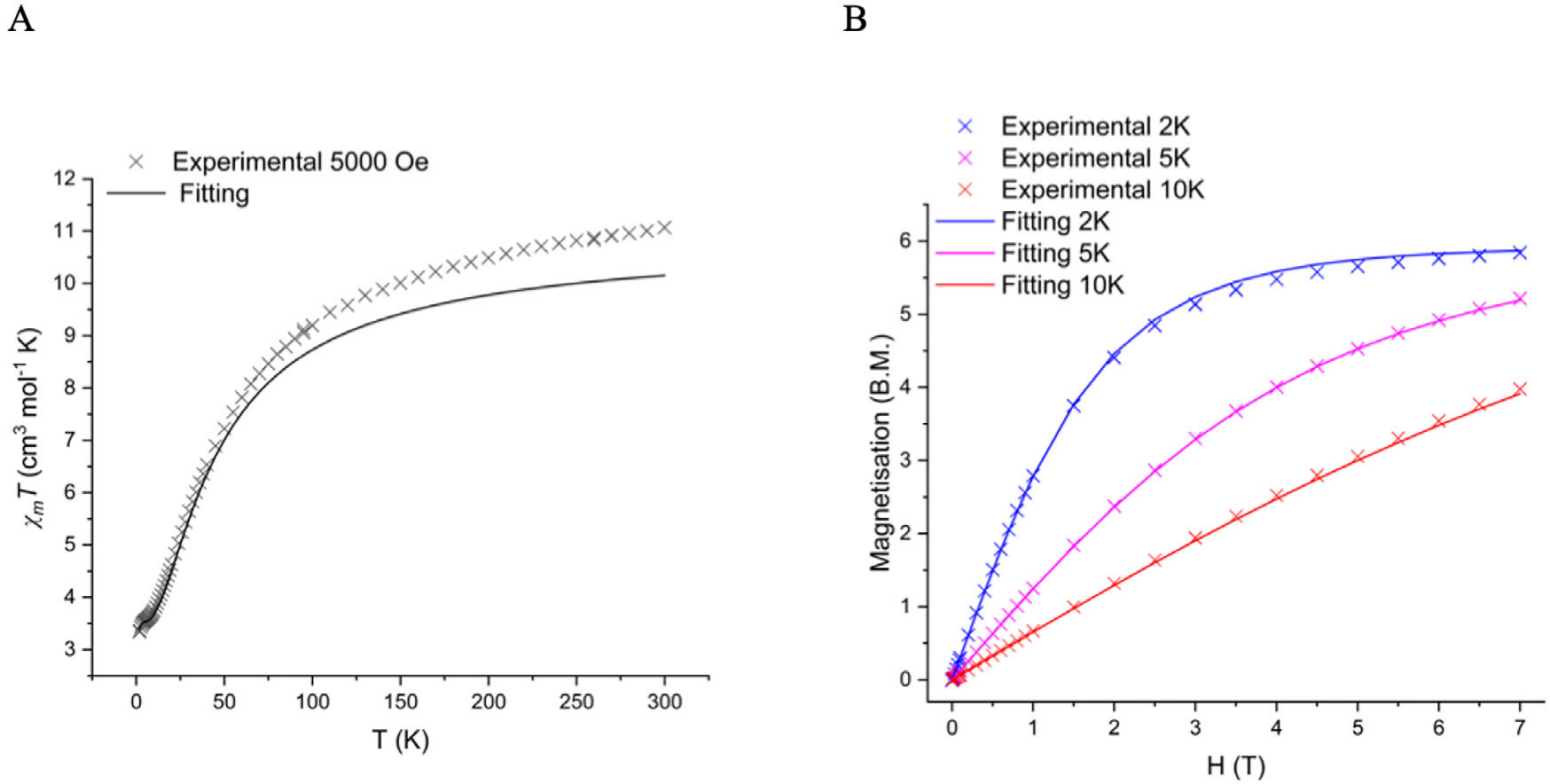
Magnetic studies of **5‐Zn**. (A) *χ_M_T*(*T*) at 5000 Oe. (B) *M*(*H*) measured at 2, 5, and 10 K. In (a) and (b), experimental data are shown as crosses, simulation using the parameters in Table 3 is shown as a line.

The magnetic susceptibility (*χ*_M_) as a function of temperature was studied for **5‐Zn** in the temperature range of 2 to 300 K under an external field of 5000 Oe. Additionally, the magnetization (*M*) was measured as a function of field at temperatures of 2, 5, and 10 K. The experimental *χ*_M_ *T* value at room temperature is 11.07 cm^3^ mol^−1^ K, which is slightly above the expected value of 10.92 cm^3^ mol^−1^ K. As the temperature decreases, the *χ*_M_ *T* value falls (Figure 10a), which is consistent with the presence of antiferromagnetic interactions between the metal centers, as expected compared to the first heterometallic wheel **1**. The plot of *M*(*H*) at 2, 5, and 10 K reaches saturation at 2 K but not at higher temperatures (Figure 10b). The observed saturation magnetization indicates an *S *= ^3^/_2_ ground state. The *χ*_M_ *T*(*T*) and *M*(*H*) data were fit simultaneously using the PHI software [48], assuming the single isomer with Zn(II) centers at 1,5‐positions, giving the parameters summarized in Table 3. The fit for *M*(*H*) is excellent, but there is a discrepancy at higher temperatures for *χ*_M_ *T*(*T*).

For **5‐Co**, the *χ*_M_ *T* value is 16.70 cm^3^ mol^−1^ K at 300 K, which is higher than expected for a noninteracting arrangement of six Cr(III) centers (*S* = ^3^/_2_, *g* = 1.97) and two octahedral Co(II) centers (*S* = ^3^/_2_, *g* = 2, and *L*_eff_ = 1), which would give a *χ*_M_ *T* value of 15.17 cm^3^ mol^−1^ K. As the temperature decreases, the *χ*_M_ *T* value also decreases, indicating the presence of antiferromagnetic interactions between the metal centers, consistent with the observation made in **1‐Co** (Figure S9a). The magnetization (*M*) increases linearly with the applied magnetic field at temperatures of 2, 5, and 10 K (Figure S9b). Considering the first‐order orbital contribution to the magnetic moment of octahedral Co(II), we did not attempt to fit these data.

### 2.6 Electron Paramagnetic Resonance (EPR)

**FIGURE 11:**
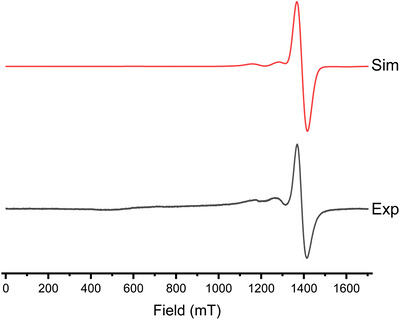
EPR spectrum of a polycrystalline sample of **4** at Q‐band (34.006 GHz) at 5 K. Experimental data are shown in black; the simulation in red uses parameters in Table 4.

**FIGURE 12:**
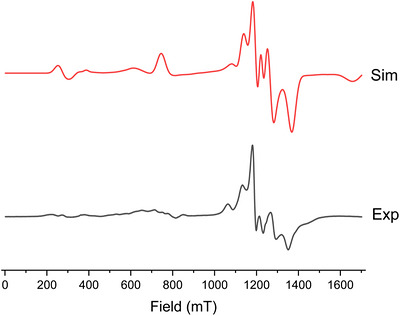
EPR spectrum of a polycrystalline sample of **5‐Zn** at Q‐band (33.900 GHz) at 5 K. Experimental data are shown in black; the simulation in red uses parameters in Table 4.

The continuous wave Q‐band (≈34 GHz) EPR spectra of **4** and **5‐Zn** were obtained at various temperatures (5, 10, 20, and 50 K, Figures 11 and 12, respectively). For **4** at 5 K, a peak is observed at 1391 mT with a *g* value = 1.75. Weaker resonances are seen at 1160 and 1315 mT (Figure 11). The spectrum is consistent with an *S *= ^1^/_2_ ground state with a low‐lying excited state with *S* = ^3^/_2_. Variable temperature measurements confirm that the weaker features are due to an excited state, as they have gained intensity compared with the *S* = ½ ground state at 10 K before broadening at 20 K (Figure S11). The EPR results again exclude isomer **E**.

For **5‐Zn** at 5 K, resonances are observed through most of the magnetic field range, similar to those previously recorded for odd‐numbered chromium chains [49] and the single isomer **2‐Zn** [24]. The spectra are noticeably better resolved than for the multiple isomers found in **3‐Zn**. Based on the magnetic studies, the spectra were simulated with two *S *= ^3^/_2_ states for the two {Cr_3_} chains present (Figure 12), and with a sharp feature at *g* = 1.97 due to the *S* = ½ excited states. Variable‐temperature measurements show all features broadening even at 10 K (Figure S12).

The giant spin approximation (GSA) was used to simulate the 5 K EPR spectra using the Easyspin [50] toolbox in Matlab [51], giving the parameters in Table 4. The higher temperature spectra were not simulated as we were using the GSA, and the main change is the broadening of the resonance features.

**TABLE 4:** Spin Hamiltonian parameters used to simulate the EPR spectra of **4** and **5‐Zn**.

Compound	*S*	Weight	*g*	*D* (strain)^a^	*E* (strain)^a^	*H*‐Strain^a^	Linewidth^b^
**4**	^1^/_2_ ^3^/_2_	0.90 0.10	1.76, 1.71 1.86, 1.81	N/A 0.11	N/A 0.001	0.04 0.07	10 30
**5‐Zn**	^3^/_2_ ^1^/_2_	0.98 0.02	1.98 1.98	0.288 (0.047) N/A	0.059 (0.003) N/A	___ ___	20 20

^a^ Units in cm^−1^

^b^ Units in mT.

### 2.7 Nuclear Magnetic Resonance (NMR) of 5‐Co

**FIGURE 13:**
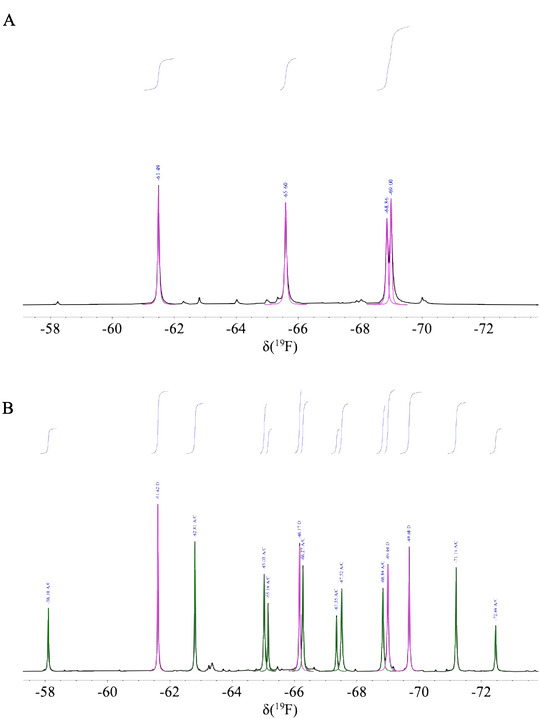
The ^19^F‐NMR spectra of compounds **5‐Co** (A) and **3‐Co** (B) were obtained at 471 MHz in d^6^ acetone at 20°C. The spectra show resonances corresponding to an isomer where the Co(II) centers are located at the 1,5‐positions on the ring, indicated in purple for both **5‐Co** and **3‐Co**. For **3‐Co**, resonances are observed for another isomer, where the Co(II) centers are positioned at the 1,2‐positions on the ring, indicated in green [25].

The **5‐Co** exhibits paramagnetically shifted NMR spectra due to the fast electron spin relaxation associated with the octahedral Co(II) metal ion. Among the possible options for NMR, ^1^H‐NMR and ^19^F‐NMR, the latter is more useful for observing ‐CF_3_ groups than the aromatic protons of BTFB^–^ (Figure S12), making ^19^F‐NMR more informative in this context. The presence of four well‐resolved resonances with similar integration values is only consistent with a single isomer of **5‐Co**, with the Co(II) centres located at the 1 and 5 positions within the ring. The four resonances are observed at −61.49, −65.60, −68.86, and −69.00 ppm (Figure 13a). These results confirm the observations made for **3‐Co**, which contains two isomers; four of the resonances in those ^19^F‐NMR spectra were attributed to the 1,5‐isomer for **3‐Co** (Figure 13b), which had similar chemical shifts [25].

## 3 Discussion

Previously, using BTFB^−^ with Pr_2_NH^+^ in the direct synthesis resulted in the formation of {Cr_6_M_2_} **3‐M**, where the positions of the divalent metals were disordered. In contrast, using O_2_C^t^Bu^−^ and Me_4_N^+^ led to forming **2‐M**, with the divalent metals localized at the edges of the octagon in positions 1 and 5. In both these cases, the divalent 3d‐metals showed no discrimination, with cobalt(II), nickel(II), and zinc(II) behaving identically. The combination of BTFB^−^ and Me_4_N^+^ in the direct synthesis yielded an unexpected result: the formation of {Cr_5_Ni_3_} **4**, while for cobalt(II) and zinc(II), the expected single isomer {Cr_6_M_2_} rings were formed. We have used a combination of CID‐MS and magnetic measurements to show **4** forms as a single isomer.

Explaining these observations requires some consideration of the relative energy terms present in the formation of any heterometallic ring. Given the complexity of the structures, this discussion begins by considering metal‐ligand, Coulombic, and hydrogen bonding interactions.

We now have five different cases of {Cr^III^_8‐x_M^II^_x_} for *x* = 0, 1, 2, and 3; for *x* = 2, we have cases where the divalent metal sites are ordered at the 1,5‐positions of the ring and disordered with the 1,5‐isomer, one of two found. Each metal site in these compounds is six‐coordinate, with two bonds to fluoride and four bonds to oxygen donors from carboxylates. If we begin with *x* = 0, we have neutral [CrF(O_2_CR)_2_]_8_ **6**, which was first made with R = *^t^* Bu [52], but which has recently been made with other carboxylates [53]. When we move to *x* = 1, we lose two Cr^III^‐F and four Cr^III^‐O bonds, gaining two M^II^‐F bonds and four M^II^‐O bonds, and also a Coulombic attraction between the anionic ring and a cation, *E*_Coul_, and an energy term due to N‐H….F hydrogen bonding, *E*_N‐H…F_. Therefore, we will preferentially form **1‐M** over **6** if:

(1) $$2E_{\mathrm{Cr}-F}+4E_{\mathrm{Cr}-O}<2E_{M-F}+4E_{M-O}+E_{\mathrm{Coul}}+E_{N-H\text{…}F}$$

where the terms are all expressed as formation energies for the relevant bonds/interactions. For most carboxylates, this balance seems to favor the formation of **1‐M**; however, we frequently see the formation of **6** as a byproduct.

If we compare the formation of **1‐M** and **3‐M**, then we have a very similar energy balance—we have one less trivalent Cr site and one more divalent M site. For a weakly binding carboxylate such as BTFB (the p*K*_a_ for BTFBH is 3.34) [44], the value of *E*_Cr‐O_ and *E*_M‐O_ will both be smaller, and the energy gap between them may be smaller. The Coulombic term will be bigger, as now we have a dianionic ring binding to cations. It is also probable that the *E*_N‐H…F_ term will be bigger. Therefore, changing to a weakly nucleophilic carboxylate may lead to {Cr^III^_6_M^II^_2_} rings. We stress: this is post‐rationalization; we did not expect the formation of **3‐M**.

**FIGURE 14:**
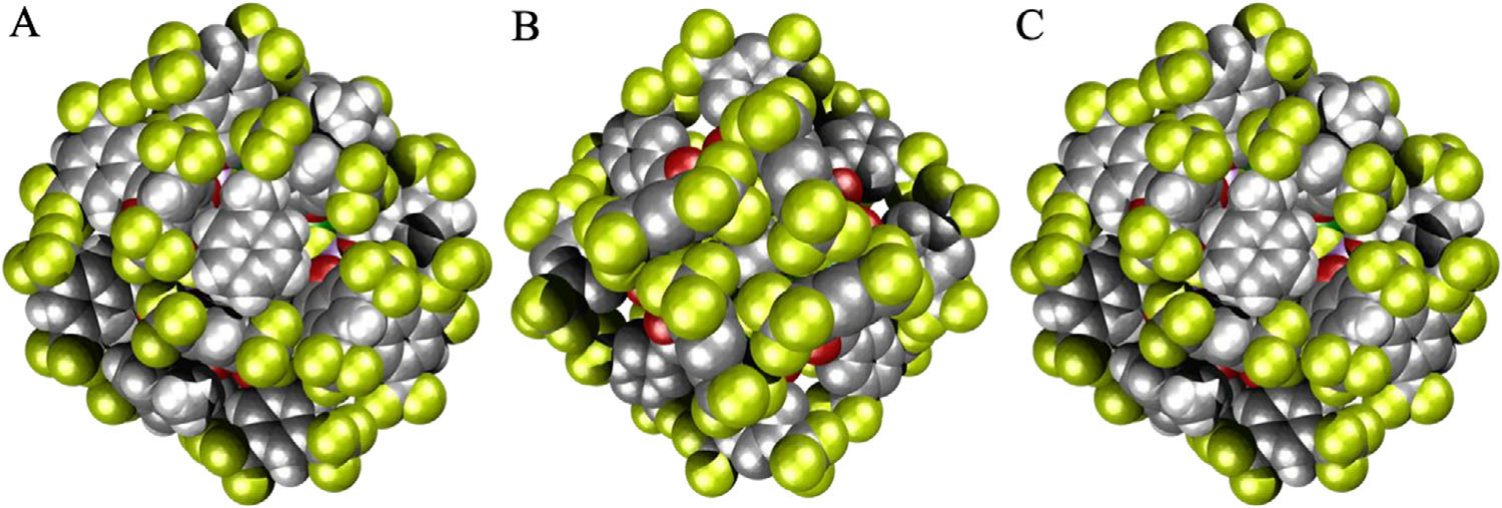
Space‐filling representations of (A) **4**, (B) **3‐Ni**, (C) **5‐Co**. The toluene molecule can be seen at the centre of the view in (A) and (C). Colors as Figure 3.

The curiosity arises from the role of the NMe_4_^+^ cation in **2‐M**, **4**, and **5‐M**; in Equation (1), we lose the *E*_N‐H…F_ term, which might lead to the prediction of the formation of **6** or at most **1‐M**. We see **6** as a byproduct, but we see the formation of the dianionic and, for **4**, a trianionic ring. This suggests a further energy term that we have neglected for this specific cation. When we examine the crystal structure of **4** closely and compare it with **3‐M**, the NMe_4_^+^ cation in **4** fits neatly into a pocket formed by the aryl‐groups of four BTFB carboxylates (Figure 14a) with nearest contacts between the methyl carbons and the centroid of the aryls of 3.22(2) Å. There are also C─H….F contacts to the bridging fluorides at around 3.32(2) Å. If we compare this with **3‐Ni** (Figure 14b), the ^n^Pr_2_NH_2_^+^ cation is encapsulated within the ligand sheath, and the nearest contacts between cation carbons and the centroid of the aryls are 3.715(8) Å. Finally, for **4** and **5‐M**, we see a toluene molecule placed above the NMe_4_^+^ cation.

This interaction between the BTFB ligands and the cation affects the orientation of these ligands. In **4**, the aryls are tipped away from the cavity, with the mean plane between the aryl rings and the mean plane of the eight metals around 101°. In **2‐Ni**, the same plane is 85° with the rings tipped towards the cavity. Therefore, this cation….aryl interaction is the term we are missing in Equation (1). In our previous paper [25], we discussed the inter‐ligand interactions; we do not think they can play a large part here, as the same sixteen carboxylates are found in both structures. The final discussion for the present results concerns why we see **4** but **5‐Co** and **5‐Zn**. If we return to Equation (1), we can see that the bond dissociation energies for the divalent metal are important; this term is larger for Ni(II) than for Co(II) or Zn(II), and we believe this indicates that we are close to the balance between the terms on each side of this equation.

This does leave one unanswered question, which is why NMe_4_^+^ leads to {Cr^III^_6_M^II^_2_} rings with pivalate as the carboxylate.

The single isomer seen for the {Cr^III^_5_Ni^II^_3_} ring is unexpected, and required a combination of magnetism, EPR spectroscopy, and, particularly, CID‐MS to establish. As seen for other heterometallic rings, there is a preference to keep the different metals as far apart as possible. For example, in centered six‐metal rings, Saalfrank et al. found that for {M_2_M’_4_} rings the 1, 4‐isomer occurred and for {M_3_M^’^_3_} rings the 1,3,5‐isomer was found. In the many 3d‐4f rings [4, 5, 6, 7, 8, 9, 10, 11, 12] alternation is always found, sometimes alternating one 3d‐metal and one 4f‐metal [4, 5, 8, 9], or alternating a dimetallic unit of one metal with a monometallic unit of the other [6, 7]. For **4** complete alternation is impossible, as we have unequal numbers of each metal. The 1,3,6‐isomer keeps the two Cr^III^ units as close in size as possible (two dimetallic and one monometallic units) while alternating with the Ni^II^ sites.

## 4 Conclusions

The composition of **4** is confirmed through elemental analysis and mass spectrometry. X‐ray diffraction (XRD) analysis cannot distinguish between the five different possible isomers. Additionally, there are three ammonium cations: two are positioned above and below the metal plane, while the third cation is located outside the ring. Magnetic and EPR studies for **4** indicate the presence of a single isomer and give unambiguous evidence that there must be an odd‐number of nickel sites in the ring. When the same chemistry is performed with Co(II) or Zn(II), we see a {Cr^III^_6_M^II^_2_} ring. Through a combination of magnetic, EPR spectroscopy, and CID‐MS, we can conclude that a single isomer is formed, which has the divalent sites at the 1, 3, and 6 positions of the metal octagon.

We propose that BTFBH acts as a stronger acid and functions as a weaker ligand compared to pivalate, with p*K*_a_ values of 3.34 (predicted) for BTFBH and 5.03 for pivalate [24]. The synthesis of the **3‐M** motif employs a template, Me_4_N^+^, which binds more weakly to the assembling ring than R_2_NH_2_^+^. This gives an empirical correlation with the use of BTFBH so that overall, a weaker carboxylate ligand and weaker H‐bonding by the templating ion lead to the incorporation of three Ni(II) centers and, hence, a higher formal negative charge on the ring.

## Conflicts of Interest

The authors declare no conflicts of interest.

## Supporting information




**Supporting File**: chem70991‐sup‐0001‐SuppMat.docx.
