# Supplementary material for: A Novel Heterometallic Ring {Cr5Ni3} and New {Cr6Co2} and {Cr6Zn2} Rings
Source: Chemistry. 2026 Apr 16;32(24):e70991. doi: 10.1002/chem.70991 (PMC13290412; doi:10.1002/chem.70991)
Supplement: Supplementary file 1 — Supporting File: chem70991‐sup‐0001‐SuppMat.docx. [file CHEM-32-e70991-s001.docx]

**Supplementary Information**

**A novel heterometallic ring {Cr_5_Ni_3_} and new {Cr_6_Co_2_} and {Cr_6_Zn_2_} rings**

Abdulelah Alsuhaymi,^1,2^ Niklas Geue,^1,3^ Adam Brookfield,^1^ Grigore A. Timco,^1^ Selena J. Lockyer,^1^ Gareth D. Smith,^1^ George F. S. Whitehead,^1^ Perdita E. Barran,^1,3^ David Collison^1^ and Richard E. P. Winpenny.^1^*

^1^Department of Chemistry, The University of Manchester, Oxford Road, Manchester M13 9PL, UK.

^2^Department of Chemistry, Faculty of Science, Islamic University of Madinah, Soultana Road, Madinah 42351, Saudi Arabia.

^3^Michael Barber Centre for Collaborative Mass Spectrometry, Manchester Institute of Biotechnology, Department of Chemistry, The University of Manchester, 131 Princess Street, Manchester, M1 7DN, UK.

**Contents**

General 2

Chemical synthesis 3

Additional mass spectrometry figures 3

X-ray crystallography 6

Ion mobility and collision-induced dissociation mass spectrometry 9

Magnetic studies 11

EPR spectra 12

NMR spectra 13

Additional references 13

**General**

**Chemicals**

All reagents and solvents were bought from Sigma-Aldrich and used without further purification. In the synthesis, the source of amine was tetramethylammonium hydroxide pentahydrate, and the acid source was BTFBH supplied by Fluorochem.

**Instrumentation**

The elemental data was obtained at the Microanalytical Service at the University of Manchester for organic analysis (Carbon, Hydrogen and Nitrogen) by using Thermo Flash 2000‌ elemental analyser and for metals analysis by using Thermo iCap 6300 inductively coupled plasma optical emission spectroscopy (ICP-OES). The mass spectrometry data was acquired at the Mass Spectrometry Service at the University of Manchester, utilising electrospray ionisation (ESI) as the technique. ESI analysis was conducted using Thermo Q-Exactive (Orbitrap with a quadrupole at the front end), and Thermo Ultimate 3000 UHPLC was used to introduce the sample into the mass spectrometer. Nuclear magnetic resonance (NMR) spectra were recorded using a Bruker AVIII HD 500 in the Chemistry NMR facility at the University of Manchester. The instrument was equipped with a 5 mm BBO Prodigy nitrogen cooled probe operating at nominal ^1^H and ^19^F Larmor frequencies of 500.19 MHz and 470.65 MHz respectively. ^19^F experiments were acquired with an interpulse relaxation delay of 20 s to ensure the experiment was quantitative. Electron paramagnetic resonance (EPR) spectra were obtained using a Q-band, with data collected on a Bruker E500 spectrometer equipped with an ER 5106 QT resonator (Q-band, ca. 34 GHz). All EPR measurements were performed on frozen solutions at a concentration of 10 mM using HPLC-grade toluene. The magnetic measurements were conducted using a Quantum Design MPMS3 SQUID magnetometer. Both the EPR and magnetic measurements took place at the National Service for Electron Paramagnetic Resonance Spectroscopy at the University of Manchester.

**Synthesis**

**Preparation of [Me_4_N][(Me_4_N)_2_Cr_5_Ni_3_F_8_(BTFB)_16_]** **4**

The following chemicals were mixed in an open Teflon^®^ flask: 1,2-dichlorobenzene (6 g, 40.81 mmol), tetramethylammonium hydroxide pentahydrate (1.08 g, 5.96 mmol), BTFBH (10 g, 38.74 mmol), chromium(III) fluoride tetrahydrate (2 g, 11.05 mmol), and basic nickel(II) carbonate (0.50 g, 1.64 mmol). The mixture was heated to 140 °C and stirred for 50 hours. After cooling to ambient temperature, the reaction mixture was transferred to a round-bottom flask, and MeCN (35 mL) was added. It was then stirred for 2 hours for precipitation. The green precipitate was filtered and washed with MeCN (3 × 25 mL). The green residue was then extracted with acetone (75 mL). Toluene (25 mL) was added to the extracted filtrate before evaporating the solvents using a rotary evaporator. The green residue was washed with toluene (about 50 mL), then hexane (25 mL), and dried in the air. Yield 4.01 g (36.82 % based on Cr), EA calculated (%) for C_156_H_84_Cr_5_F_104_N_3_Ni_3_O_32_; Cr 5.28, Ni 3.58, C 38.05, H 1.72, N 0.85; found (%): Cr 5.33, Ni 3.14, C 37.83, H 1.48, N 0.75. ES-MS (sample dissolved in THF and run in MeOH) in the negative mode, *m*/*Z*: 2387.82 [**4** − 2 Me_4_N]^2−^, 4849.68 [**4** – Me_4_N]^−^.

**Preparation of [Me_4_N][(Me_4_N)Cr_6_Zn_2_F_8_(BTFB)_16_] 5-Zn**

Following the same procedure as **4** but using basic zinc carbonate instead of nickel carbonate (0.45 g, 0.91 mmol). Yield 3.08 g (34.45 % based on Cr), EA calculated (%) for C_152_H_72_Cr_6_F_104_N_2_O_32_Zn_2_: Cr 6.42, Zn 2.69, C 37.59, H 1.49, N 0.58; found: Cr 6.28, Zn 2.77, C 37.51, H 1.50, N 0.59. ES-MS (sample dissolved in THF and run in MeOH) in the negative mode, *m*/*Z*: 2353.77 [**5-Zn** − 2 Me_4_N]^2−^, 4782.19 [**5-Zn** – Me_4_N]^−^.

**Preparation of [Me_4_N][(Me_4_N)Cr_6_Co_2_F_8_(BTFB)_16_] 5-Co**

Following the same procedure as **4** but using cobalt(II) carbonate hydrate instead of nickel carbonate (0.50 g, 4.20 mmol). Yield 2.45 g (27.46 % based on Cr), EA calculated (%) for C_152_H_72_Co_2_Cr_6_F_104_N_2_O_32_: Cr 6.44, Co 2.43, C 37.69, H 1.50, N 0.58; found: Cr 6.19, Co 2.53, C 37.66, H 1.48, N 0.59. ES-MS (sample dissolved in THF and run in MeOH) in the negative mode, *m*/*Z*: 2347.78 [**5-Co** − 2 Me_4_N]^2−^, 4769.61 [**5-Co** – Me_4_N]^−^.

**Additional mass spectrometry figures**

**Figure S1**. Full experimental ESI-MS for **4** in the positive mode.

**Figure S2**. Full experimental ESI-MS for **4** in the negative mode.

**Figure S3**. Full experimental ESI-MS for **5-Zn** in the positive mode.

**Figure S4**. Full experimental ESI-MS for **5-Co** in the positive mode.

**Figure S5**. Full experimental ESI-MS for **5-Zn** in the negative mode.

**Figure S6**. Full experimental ESI-MS for 5-Co in the negative mode.

# X-ray Crystallography

# Data collection: X-ray diffraction data for compound 4, 5-Zn and 5-Co were collected using a dual wavelength Rigaku FR-X rotating anode diffractometer using MoKα (λ = 0.5889 Å) for 4 and CuKα (λ = 1.54146 Å) radiation for 5-Co and 5-Zn, equipped with an AFC-11 4circle goniometer, VariMAX^TM^ microfocus optics, a Hypix-6000HE detector and an Oxford Cryosystems 800 plus nitrogen flow gas system, at a temperature of 100 K. Data were collected and reduced using CrysAlisPro v43.^1^ Absorption correction was performed using empirical methods (SCALE3 ABSPACK) based upon symmetry-equivalent reflections combined with measurements at different azimuthal angles.

# Crystal structure determination and refinements: The crystal structures were solved and refined against all F^2^ values using the SHELX and Olex2 suite of programmes.^2,3^ Coordinates and anisotropic displacement parameters for all non‑hydrogen atoms were freely refined. Hydrogen atoms were constrained to idealised positions with the coordinates refined to ride with the parent atom. Hydrogen isotropic atomic displacement parameters were constrained to ride with the parent atom with an appropriate multiplier for the hybridisation.

# Crystallographic data have been deposited at the Cambridge Structural Database with CCDC numbers 2456172 – 2456174.

**Table S1**. Crystallographic data for **4, 5-Co** and **5-Zn**.

| Identification code | **4** | **5-Co** | **5-Zn** |
| --- | --- | --- | --- |
| Empirical formula | C_198_H_132_Cr_5_F_104_N_3_Ni_3_O_32_ | C_202.26_H_129.44_Co_2_Cr_6_F_104_N_2_O_32_ | C_203.63_H_131_Cr_6_F_104_N_2_O_33_Zn_2_ |
| Formula weight | 5477.19 | 5505.52 | 5536.40 |
| Temperature/K | 293(2) | 100.00(10) | 100.00(12) |
| Crystal system | tetragonal | monoclinic | monoclinic |
| Space group | *P*4/*ncc* | *P*2/*n* | *C*2/*c* |
| *a*/Å | 25.82541(12) | 34.4682(7) | 36.8311(8) |
| *b*/Å | = *a* | 19.0957(3) | 36.9721(7) |
| *c*/Å | 35.0083(2) | 36.8469(7) | 34.6577(8) |
| *α*/° | 90 | 90 | 90 |
| *β*/° | 90 | 104.886(2) | 101.299(2) |
| *γ*/° | 90 | 90 | 90 |
| Volume/Å^3^ | 23348.8(3) | 23438.5(8) | 46279.5(18) |
| *Z* | 4 | 4 | 8 |
| *ρ*_calc_g/cm^3^ | 1.558 | 1.560 | 1.589 |
| *μ*/mm^-1^ | 0.561 | 4.620 | 3.814 |
| *F*(000) | 10948 | 10988 | 22102 |
| Crystal size/mm^3^ | 0.345 × 0.325 × 0.316 | 0.290 × 0.257 × 0.088 | 0.316 × 0.181 × 0.141 |
| Radiation | MoKα (λ = 0.6889) | Cu Kα (λ = 1.54184) | Cu Kα (λ = 1.54184) |
| Reflections collected | 310209 | 101882 | 44692 |
| Independent reflections | 13928 | 37630 | 44692 |
| Data/restraints/parameters | 13928/4225/1559 | 37630/22707/4115 | 44692/36801/5078 |
| Goodness-of-fit on *F*^2^ | 1.306 | 1.335 | 1.055 |
| Final *R* indexes [*I*>=2*σ* (*I*)] | R_1_ = 0.0975,  wR_2_ = 0.3000 | R_1_ = 0.1180,  wR_2_ = 0.3440 | R_1_ = 0.0972,  wR_2_ = 0.2820 |
| Final *R* indexes [all data] | R_1_ = 0.1135,  wR_2_ = 0.3215 | R_1_ = 0.1646  wR_2_ = 0.3759 | R_1_ = 0.1213,  wR_2_ = 0.3016 |
| Largest diff. peak/hole /  e Å^-3^ | 1.022/-0.740 | 1.025/-0.759 | 1.212/-0.821 |

**
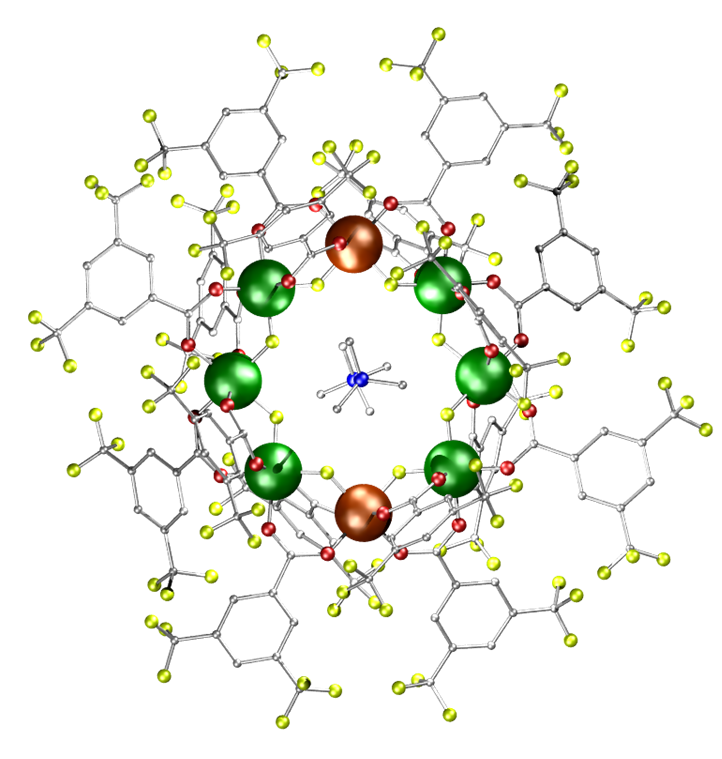
**

**Figure S7**. The structure **5-Zn** in the crystal. Colours as Figure 3, plus Zn, brown.

Ion Mobility and Collison-induced Dissociation Mass Spectrometry

**Sample Preparation**

Samples were prepared at 200 µM in 4:1 toluene/methanol.

**Ionisation Conditions**

Samples were ionized and transferred to the gas phase with an nESI source and were sprayed from borosilicate glass capillaries (World Precision Instruments). The latter were pulled on the Flaming/Brown P-2000 laser puller (Sutter Instrument Company). The capillary voltage (1.5 - 2.5 kV) was applied through a platinum wire (diameter 0.125 mm, Goodfellow) inserted into the nESI capillaries. Source temperature was set to 30 °C (IM-MS Studies) and 23°C (CID-MS Studies), respectively.

**IM-MS Studies**

Ion mobility mass spectrometry (IM-MS) experiments were performed on a Select Series Cyclic IMS (Waters).^4^ After the transfer to the gas phase (Cone Voltage: 20 V, Source Offset: 10 V), [**1** – 2 NMe_4_]^2-^ was isolated in a quadrupole mass filter and subsequently injected into the cyclic ion mobility drift ring. In this region, ions were separated by using a non-uniform electric field under a constant nitrogen pressure. Travelling waves (TW, Height: 22 V) pushed the ions through the cyclic drift region. Ions travelled 185 passes in the cyclic drift ring (181 m, separation time: 4.995 s) and were subsequently transferred (Transfer Energy: 4 V) to a time-of-flight mass analyser.

**CID-MS Studies**

The Q Exactive Ultra-High-Mass-Range (UHMR) Hybrid Quadrupole-Orbitrap Mass Spectrometer (Thermo Fisher) was used for the derivation of the *E*_50_ values *via* CID-MS experiments.^5^ Target ions were *m*/*z-*isolated in a quadrupole filter, accelerated to a user-defined kinetic energy (*E*_lab_: 0–300 eV) and injected into the higher-energy C-trap dissociation cell, which contained nitrogen gas (trapping gas pressure parameter, 2.0). Non-fragmented precursor ions and fragment ions were transferred to the Orbitrap mass analyser (maximum inject time, 100 ms; resolution, 25,000).

*E*_50_ values were obtained from a method described in our previous works (see references in manuscript). Mass spectra were recorded at different collision energies, and the share of the precursor ion count, relative to the total ion count (‘survival yield’), was plotted versus the collisional energy in the centre-of-mass frame (*E*_com_). Survival yield plots were fitted with a sigmoidal Hill function (Hill1 function in OriginPro 2020b), yielding the point (*E*_50_) at which the survival yield reaches 0.5 or 50%.


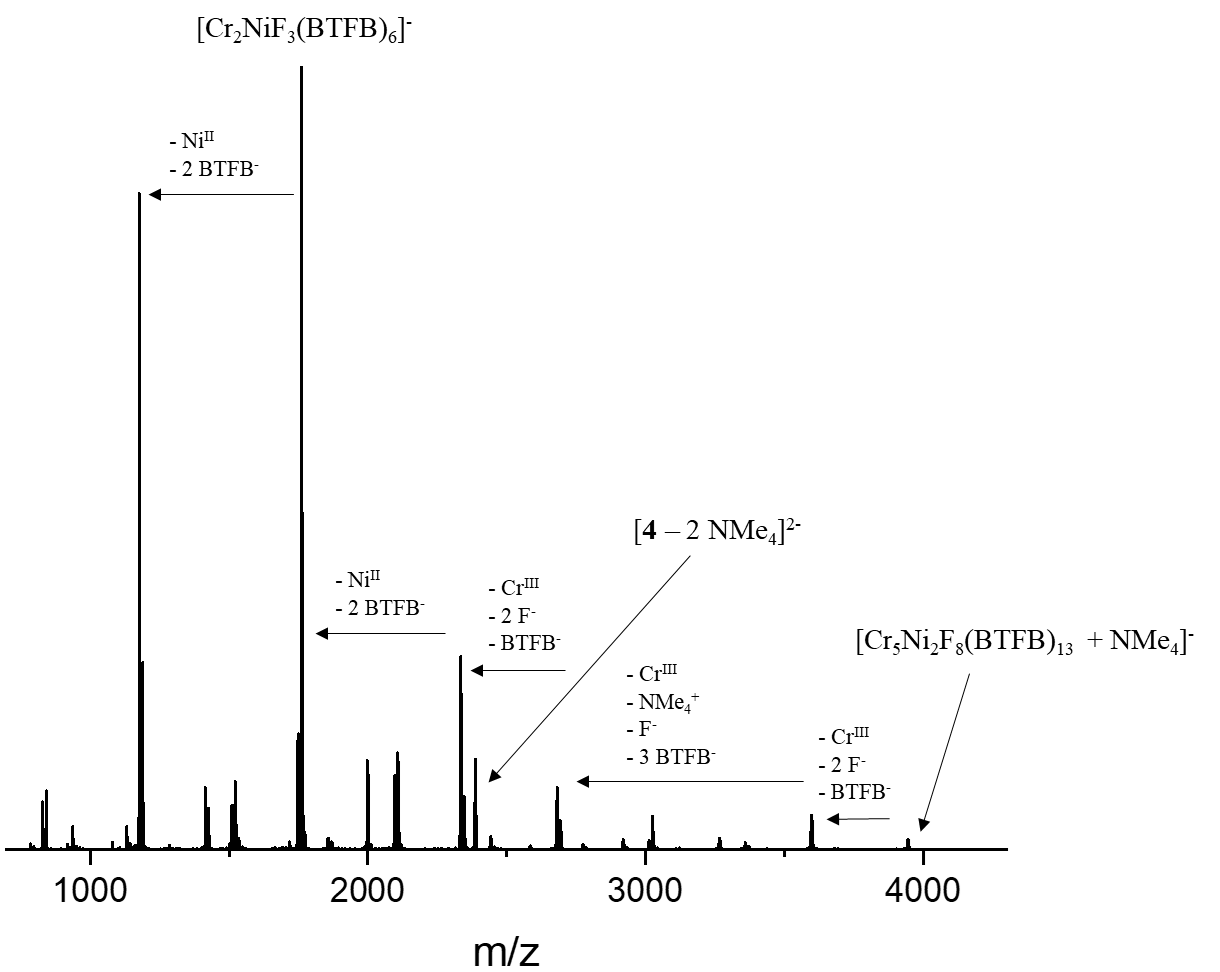


**Figure S8:** CID-MS spectrum of [**4** – NMe_4_]^2-^ (*m/z* = 2387) at collision energy in the laboratory frame *E_lab_* = 180 eV. Fragmentation channels are diverse, and the precursor ion first fragments to [Cr_5_Ni_2_F_8_(BTFB)_13_ + NMe_4_]^-^ through a charge loss. Subsequent channels are then similar to the singly charged anion, and the most dominant ones are labelled.

Magnetic studies

**Figure S9**. Fit of magnetism of **4** to isomer **A** (Figure 4). (a) χ_M_T(T) of **4** measured at 1000 Oe. (b) M(H) measured at 2, 5 and 10 K. In (a) and (b) experimental data shown as crosses. Simulation to isomer **A** (i.e. Ni^II^ at 1,2,3-positions of octagon using parameters *J*_CrCr_ = -4.48, *J*_CrNi_ = -18.23, *J*_NiNi_ = -4.61 cm^-1^, *g^Cr^* = 1.97, *g^Ni^* = 2.20.

**Figure S10**. Magnetic studies of **5-Co**. Experimental shown as crosses, (a) χ_M_T(T) of **5-Co** measured at 1000 Oe. (b) M(H) measured at 2, 5 and 10 K.

# EPR spectroscopy





**Figure S11**. The variable temperature EPR spectra of **4** at a Q-band (5.0 K: 34.006 GHz; 10 K: 34.040 GHz; 20.0 K: 34.057 GHz; 50.0 K: 34.066 GH).





**Figure S12**. The variable temperature EPR spectra of **5-Zn** at a Q-band (5.0 K: 33.900 GHz; 10 K: 33.898 GHz; 20.0 K: 33.915 GHz; 50.0 K: 33.927 GH).

# NMR Spectroscopy

**Figure S13**. ^1^H-NMR spectrum of **5-Co** measured at 500 MHz in d^6^ acetone at 20 °C.

**Additional References**

1. Rigaku Oxford Diffraction, (2024), CrysAlisPro Software system, version 1.171.43.116a, Rigaku Corporation, Wroclaw, Poland.
2. (a) G. M. Sheldrick, G. M. SHELXT – Integrated space-group and crystal structure determination. *Acta Cryst*. *A* **2015**, *71*, 3-8; (b) G. M. Sheldrick. Crystal structure refinement with SHELXL. *Acta Cryst. C* **2015**, *71*, 3-8.
3. O. V. Dolomanov, L. J. Bourhis, R. J. Gildea, J. A. K. Howard, H. Puschmann. OLEX2: a complete structure solution, refinement and analysis program. *J. Appl. Cryst*. **2009**, *42*, 339-341.
4. K. Giles, J. Ujma, J. Wildgoose, S. Pringle, K. Richardson, D. Langridge, M. A. Green, Cyclic Ion Mobility-Mass Spectrometry System. *Anal. Chem.* **2019**, *91*, 8564–8573.
5. K. L. Fort, M. Van De Waterbeemd, D. Boll, M. Reinhardt-Szyba, M. E. Belov, E. Sasaki, R. Zschoche, D. Hilvert, A. A. Makarov, A. J. R. Heck. *Analyst* **2018**, *143*, 100–105.
